# Translating sensory input into motor output: A novel tool in tooth morphology

**DOI:** 10.6026/973206300212191

**Published:** 2025-07-31

**Authors:** Vaishnavi Jammula, Soumya Anandan, Aditasaivam V., Sonali D., Thota Deepavi

**Affiliations:** 1 Department of Oral Pathology and Microbiology, Sri Ramachandra Dental College, Sri Ramachandra Institute of Higher Education, Chennai, India

**Keywords:** Tooth morphology, sensory skills, skill development

## Abstract

Mastering tooth morphology requires sensory-motor skill development through careful planning and coordination. Carving teeth in wax models enhance hand skills and comprehension of tooth structure. Repetition and practice reinforce skills and concepts. With dedicated practice, students can develop expertise in identifying anatomical landmarks and applying knowledge in clinical settings. This skill development is essential for accurate tooth identification and treatment.

## Background:

To identify tooth abnormalities, it is essential to first understand normal tooth morphology. Skill development relies on the sensory system, which converts signals into motor skills through proper planning and coordination, embedding them in the subconscious mind [1]. The sensory division of the nervous system collects information from the external world and monitors internal changes. Sensation involves the conscious perception of sensory data reaching the brain. Sensory receptors, such as mechanoreceptors (Pacinian corpuscles, Meissner's corpuscles, Merkel's discs and hair end organs), detect stimuli and convert them into neural signals [2]. Sensory transduction refers to the conversion of environmental signals into neural signals by these receptors [2]. The somatic motor system, made up of various nervous system components, manages the execution, planning, coordination and adjustment of body movements [3]. Tooth morphology is crucial for understanding the detailed structure of teeth, aiding in distinguishing their features. A strong grasp of the subject is essential, as "the eyes cannot see what the mind does not know". Tooth morphology provides insights into grooves and fossae, which are key to crushing, grinding, and mastication. Typically, carving replicates the natural tooth size, but this study explores carving at twice and three times the original size. Proportional carving in three sizes enhances hand skills and deepens understanding of tooth morphology. Accurate dimensions are vital for proper occlusion and the curve of Spee [4]. In the first year of dental education, students concentrate on developing strong foundational skills in tooth carving. During the second year, they apply this knowledge to gypsum models by preparing cavities and filling them with modelling wax, then sculpting the morphology to accurately replicate natural teeth. These initial two years form the pre-clinical phase of their training.

As students' progress to their third year, they enter a transitional phase that combines pre-clinical instruction with clinical practice. Before engaging with real patients, they simulate procedures on typodonts, mastering the use of amalgam for restorations. After completing these fillings, they carve them to align with the natural morphology of teeth. However, many students struggle to recall the intricate details of tooth morphology learned in their first year, which hampers their clinical performance. Consequently, this study aims to achieve three primary objectives. Firstly, it seeks to enhance motor skills by improving manual dexterity through a novel tooth-carving exercise. Secondly, it aims to assess the level of understanding among first-year students regarding the skills employed to develop motor skills. Lastly, the study aims to develop an innovative tooth-carving methodology that facilitates a deeper understanding of the anatomical landmarks of a tooth. Therefore, it is of interest to integrate this methodology in the daily practice of the undergraduate education.

## Materials and Methods:

## Materials needed:

[1] Macintosh sheet

[2] Lacron carver

[3] Wax blocks

[4] Soap

[5] Polishing cloth

[6] Metal scale

## Methodology:

## Incisors carving steps:

## Step 1: Preparation:

[1] Divide the wax block into three parts: crown and two root portions.

[2] Demarcate labial and lingual sides by dividing the block horizontally.

## Step 2: Crown portion:

[1] Divide the crown into three equal parts: incisal third, middle third and cervical third.

[2] Reduce labial portion equally, but reduce lingual side more in the incisal third.

[3] Round distal side incisal angle, while keeping mesial side sharp.

[4] Depict cingulum on lingual side in cervical third.

[5] Create depression area above cingulum, varying shape for each tooth type:

1) Central incisors: W-shaped

2) Lateral incisors: U-shaped

3) Mandibular incisors: Less prominent fossa

## Step 3: Root portion:

[1] Carve root in cone shape, with more bulk in cervical area than apical area.

[2] Ensure labiolingual width is greater than mesiodistal width. Make distal bulk more prominent in apical area to indicate quadrant affiliation.

## Canine carving steps:

## Step 1: Preparation:

[1] Divide the wax block into three parts: crown and two root portions.

[2] Demarcate labial and lingual sides by dividing the block horizontally.

## Step 2: Crown portion:

[1] Divide the crown into three equal parts: incisal third, middle third and cervical third.

[2] Reduce labial portion equally, but reduce lingual side more in incisal and middle thirds.

[3] Carve mesial slope short and sharp, and distal slope long and rounded.

[4] Depict cingulum on lingual side in cervical third.

[5] Create depression area above cingulum, depicting lingual fossa.

[6] Carve lingual ridge between two fossae.

[7] Create marginal ridges on lingual side: mesial marginal ridge and distal ridge.

## Step 3: Root portion:

[1] Carve root in cone shape, with more bulk in cervical area than apical area.

[2] Ensure labiolingual width is greater than mesiodistal width.

[3] Tilt root towards distal side in apical area, indicating quadrant affiliation.

## Premolar carving steps:

## Step 1: Preparation:

[1] Divide the wax block into three parts: crown and two root portions.

[2] Demarcate buccal and lingual sides by dividing the block horizontally.

[3] Divide the crown portion into three equal parts (occlusal one-third, two-thirds and three-thirds).

## Step 2: Crown carving:

[1] Reduce the buccal portion equally, but reduce the lingual side more.

[2] Carve the distal slope short and sharp and the mesial slope long and rounded.

[3] Draw a central groove with two triangular fossae, dividing the buccal and palatal cusps.

## Step 3: Unique features:

[1] First premolar: H-shaped groove and mesial marginal ridge.

[2] Supplementary grooves are present in premolars.

[3] Mandibular first premolar: mesiolingual developmental groove.

[4] Mandibular second premolar: Y-shaped fossa.

[5] All premolars are single-rooted, except the maxillary first premolar.

## Maxillary molar carving steps:

## Step 1: Preparation:

[1] Divide the wax block into three parts: crown and two root portions.

[2] Demarcate buccal and palatal sides by dividing the block horizontally.

[3] Divide the crown portion into three equal parts (occlusal one-third, two-thirds and three-thirds).

## Step 2: Crown carving:

[1] Draw two lines on either side (mesial and distal).

[2] Carve the oblique ridge on either side (mesiobuccal and distopalatal cusps).

[3] Mesiopalatal cusp is the largest.

[4] Add the fifth cusp (cusp of Carabelli) on the mesiopalatal cusp.

[5] Four cusps are present:

a. Two on buccal aspect (mesiobuccal and distobuccal).

b. Two on palatal aspect (mesiopalatal and distopalatal).

[6] Reduce and deepen with two triangular fossae on mesial and distal sides.

## Step 3: Root carving:

[1] Maxillary molar has three roots:

1) Two on buccal aspect (mesiobuccal and distobuccal).

2) One on palatal.

[2] Palatal roots are broader than buccal roots.

## Mandibular molar carving steps:

## Step 1: Preparation:

[1] Divide the wax block into three parts: crown and two root portions.

[2] Demarcate buccal and lingual sides by dividing the block horizontally.

[3] Divide the crown portion into three equal parts (occlusal one-third, two-thirds and three-thirds).

## Step 2: Crown carving:

[1] Reduce buccolingual width more than mesiodistal.

[2] Draw the central groove, dividing the crown into buccal and lingual aspects.

[3] Carve three cusps on buccal aspect (mesiobuccal, distobuccal, distal cusp).

[4] Carve two cusps on lingual aspect (mesiolingual, distolingual cusp).

[5] Reduce and deepen the central groove with two triangular fossae on mesial and distal sides.

## Step 3: Root carving:

[1] Mandibular molars have two roots (mesial and distal roots).

[2] Keep apical bulk more distally to denote the curve towards the distal side.

[3] Deep developmental depressions are present on the root trunk.

## Results:

The importance of tooth morphology in dental education cannot be overstated. It serves as the foundation for understanding the complex details of grooves and fossae, which are essential for the proper functioning of teeth in crushing, grinding, and chewing. Carving teeth in three different sizes helps improve hand skills, which are critical for dental professionals. These skills are directly tied to the detailed and accurate creation of teeth, which plays a significant role in achieving proper occlusion and understanding the curve of Spee. When carving a tooth, working with larger wax models allows students to grasp the finer details of tooth morphology and dimensions. This is crucial for developing a deeper understanding of the tooth's shape and function. Once students become proficient with the larger models, carving teeth in their original sizes further refines their hand skills. This progression from large to small carvings enhances both the student's technical proficiency and comprehension of the anatomy of the teeth. The development of hand skills requires continuous practice. Repetition is vital for reinforcing the concepts and techniques learned in the classroom. It is a well-established fact that passive learning, such as merely observing, is less effective than active learning, where students engage in hands-on practice. This active involvement leads to better retention and mastery of the material. In a typical week, three tooth morphology classes are held, which amounts to approximately 132 classes over 44 weeks (excluding holidays). Basic exercises such as carving simple shapes like pyramids, cuboids, and rectangles take about 9 hours to complete. Over the course of 264 hours, 9 hours are allocated to these fundamental exercises, while the remaining time is dedicated to carving 84 teeth in three consecutive sizes. For each size, carving requires 85 hours, which means each tooth takes around 3 hours to carve. By successfully completing the carving of one tooth in three hours, students will find it easier to complete all three sizes. This process solidifies their understanding and improves their ability to carve accurately. Through repetition, students will subconsciously internalize the basic principles of tooth morphology. This ongoing practice ensures that foundational concepts become second nature, allowing for better performance in the more advanced stages of dental training. Thus, continual practice and repetition are essential for mastering the intricate details of tooth morphology and carving techniques.

## Discussion:

The development of skill, especially in areas such as tooth carving and other dental procedures is deeply connected to the sensory division of the human nervous system [7]. This system plays a crucial role by converting sensory signals into motor actions, which are then processed and coordinated by the brain to produce skillful movements. The ability to carve teeth accurately and perform dental procedures is a direct result of how sensory information is collected, interpreted, and transformed into motor responses [5]. This process becomes ingrained in the subconscious through constant practice, which refines coordination and skill. The sensory division of the nervous system is responsible for gathering information about both external stimuli (*e.g.*, touch, pressure, temperature) and changes within the body itself. Sensory receptors, which include mechanoreceptors such as Pacinian and Meissner's corpuscles, are specialized cells that detect these stimuli and convert them into neural signals that the brain can interpret [2]. Sensory transduction refers to this process of converting environmental signals into neural signals, which are essential for motor coordination [6]. This sensory input allows the brain to plan, execute, and adjust bodily movements accurately. In the context of dental education, the repetition of tasks such as carving teeth helps refine motor skills and allows the fine details of tooth morphology (*e.g.*, grooves, fossa, roots, proportions) to become embedded in the brain [5]. Through consistent practice, the brain stores these details, making it easier for the dentist to recall and apply them when needed in clinical practice. As a result, the knowledge and understanding of tooth morphology become long-lasting and instinctive, forming a foundation for successful dental procedures. Additionally, working under time pressure with clear goals accelerates learning. The pressure of completing tasks within a set timeframe fosters a new, more efficient way of approaching dental procedures. This practice is not only beneficial for tooth carving but also applies to other areas in dentistry, such as amalgam carving, inlay crown preparation, tooth preparation, root canal therapy, cavity preparation, and even tooth extractions. In every sector of dentistry, as well as in other areas of life, constant practice leads to finding better, more effective ways of doing things. The connection between practice and skill is especially evident when we consider the importance of carving in dental education. If a dentist has not experienced carving a tooth during their education, they may struggle to perform tasks like composite restoration in a patient's mouth. Even a tiny flaw, such as a high-point in a restoration, can lead to issues like temporomandibular joint (TMJ) disorders. Understanding tooth shape and size is fundamental for a dentist to accurately differentiate between primary and permanent dentition, which is critical for providing proper treatment. A strong foundation in dental morphology is essential not only for the recognition of tooth structures but also for clinical applications such as diagnosis, treatment planning, and oral rehabilitation. Without a deep understanding of occlusal morphology, a dentist cannot effectively correct occlusion or achieve equilibrium in the patient's bite. This knowledge is vital for providing high-quality, functional dental care.

## Conclusion:

In summary, the development of skill through constant practice and sensory integration is essential for dentists to master the intricacies of tooth morphology and other dental procedures. Without this foundational knowledge, effective patient care and treatment cannot be achieved.
